# A qualitative meta-analysis on the lived experience of siblings of persons with mental illness

**DOI:** 10.3389/fpsyg.2026.1787699

**Published:** 2026-05-05

**Authors:** Massimiliano Sommantico, Marina Lacatena, Jacopo Postiglione, Santa Parrello

**Affiliations:** 1 Dynamic Psychology Laboratory, Department of Humanities, University of Naples Federico II, Naples, Italy; 2 Department of Humanities, University of Naples Federico II, Naples, Italy

**Keywords:** emotional impact, mental illness, need for support, personal and relational growth, qualitative meta-analysis, siblings

## Abstract

Having a brother or sister with mental illness (MI) has a profound effect on well-siblings’ health, quality of life, and relationships, and there is relatively little knowledge about their needs for information and support. The article presents a qualitative meta-analysis examining the lived experience of well-siblings of persons with MI. We explored the EBSCOHost, EMBASE, Scopus, and the Web of Science, with regards to the search terms “sibling* or brother* or sister*” AND “mental ill or mental illness or mental disease* or mental disorder*” in the abstract, AND “qualitativ* or thematic analys* or grounded theory or phenomenolog* or content analys* or narrative analys*” in any field. Using a critical-constructivist grounded theory qualitative meta-analytic approach, from 369 potentially eligible articles, we reviewed 42 qualitative studies (a total of 565 participants; 63.2% sisters) to understand the lived experience of well-siblings of persons with MI. Inclusion criteria were: (a) be peer-reviewed journal articles published until November 30, 2025; (b) be a qualitative study; (c) be in the English language; and (d) focus exclusively on describing the experience of well-siblings of persons with MI. Findings indicated that MI significantly affects well-siblings’ emotions, health, and well-being, as well as academic, working, and personal life; that well-siblings feel excluded, abandoned or treated as secondary, and have to keep their needs or feeling to themselves or have to suppress them not to burden others. Findings also indicated that well-siblings express their need of information on MI issues, as well as their need for support from significant others and from mental health practitioners. Furthermore, findings indicated that well-siblings also experienced positive changes and personal growth at different levels in facing with their brother or sister’s MI, as well as hope in the future. Finally, findings indicated that in dealing with MI, well-siblings adopt different caregiving roles, as well as different coping strategies. Giving voice to well-siblings of people with MI, this meta-analysis illuminates the challenges they experience and provides a basis for recommendations to support them in facing their emotional burden and their caregiving role.

## Introduction

According to data from the World Health Organisation (2025), nearly one in seven people worldwide – about 1.1 billion individuals – live with a mental illness (MI). MI involves a clinically significant disturbance in cognition, emotional regulation, or behavior and is often linked to distress or impairment in critical areas of daily functioning. It is also frequently associated with other challenges, such as physical illnesses, unemployment, homelessness, incarceration, and an increased risk of self-harm. Furthermore, in more developed countries, the process of deinstitutionalization – namely, the closure of psychiatric hospitals and the transition of patients to community-based services – has intensified the caregiving and support burden placed on family members of individuals with MI (Bredewold et al., 2020; Holmes and Deb, 1998; Ohaeri, 2003) which can translate into psychological consequences such as stress, feelings of loneliness, helplessness, depression, and a general reduction in well-being and physical health (Cham et al., 2022; Lin et al., 2018; Ólafsdóttir et al., 2020; Phillips et al., 2023). Usually, the primary caregiver role is assumed by parents, thus providing daily living assistance, financial and emotional support, and treatment management among others (Chen and Lukens, 2011; Karp and Tanarugsachock, 2000; Rowe, 2012; Sporer et al., 2019; Susanti et al., 2024). But, as parents age, pass on, or are unavailable for any reason, well-siblings may keep this role. This usually has a significant impact, often described as complex and longstanding, also depending on the severity of the brother or sister’s MI, on when the brother or sister’s symptoms arise (childhood, adolescence, early adulthood), on other competing caregiving roles (children, partner, aging parents), and on the nature of the interaction with mental healthcare system (Dulek et al., 2021; Hatfield and Lefley, 2005; Greenberg et al., 1999; Schaffer, 2021a, 2021b; Tharoor and Ganesh, 2015).

Several recent literature reviews on the mental health and well-being of well-siblings of individuals with severe MI (Cui et al., 2025; Heneghan et al., 2024; Izon et al., 2024; Jayasinghe et al., 2023a; Lønning et al., 2024) highlighted that research findings mainly report negative well-sibling outcomes. In particular, in their mixed-study and meta-analysis, Jayasinghe et al. (2023a) highlighted that well-siblings of people with MI significantly suffer from depressive and anxiety symptoms, experience high levels of burden, as well as low levels of well-being. Moreover, well-siblings have complex family relations, must witness frequent arguments and to mourn the brother or sister as he or she was before the onset of the MI, but also experience themselves as secondary to the ill brother or sister, “struggling with unanswered questions about the illness” (p. 6958), often step in parental-like roles, and encounter several difficulties when facing mental health services.

Focusing on well-siblings of people with eating disorders, the review of Heneghan et al. (2024) highlighted the substantial daily impact of a brother or sister’s pathology on well-siblings’ identity formation and on the way well-siblings view themselves and the world, as well as on their schoolwork, with reported higher levels of depression and anxiety and poorer quality of life. Furthermore, poorer family and sibling communication and relationships were reported, with the feeling of being “sidelined” (p. 385), receiving less time and attention than their sibling with an eating disorder. Finally, well-sibling participants, especially sisters, reported significant changes in how they view food, food choices, and eating behaviors, as well as difficulties expressing their emotions and needs, especially not to burden others (particularly parents).

The narrative review by Izon et al. (2024), focusing on the psychosocial stressors of well-siblings of people with experience of psychosis, firstly evidenced the strong impact of social stigma on the way well-siblings look at MI, with consequent feelings of embarrassment and loneliness. Furthermore, the review highlighted the key role well-siblings play in caring for their ill brother or sister, their frequent parentification, and their avoidance of caregiving responsibilities by distancing themselves. The emotions expressed towards siblings with MI are also very varied, ranging from compassion and empathy to frustration and exhaustion. The review also reported both stress and burden related to caregiving responsibilities, as well as the need for formal and informal support.

By comparing with well-control individuals, Lønning et al. (2024) reported negative well-sibling outcomes in terms of more mental health problems and lower levels of well-being, such as deterioration in academic performance, impaired functioning in autonomy, occupation, cognition, interpersonal relationships, workability, practical housework, social activities, and relations, higher rates of psychiatric disorders, more emotional distress, and poorer sibling relationships. The review also found that younger well-siblings reported higher emotional burden than brothers, similar to unmarried well-siblings. In contrast, higher education in well-siblings was associated with less emotional and objective burden, as well as with better mental health knowledge.

Finally, the review by Cui et al. (2025) also identified several forms of well-siblings’ involvement in caring for a brother or sister with MI, varying from emotional, financial, social, and instrumental care and depending on the severity of the symptomatology and on the nature of past and actual sibling and family relationships. Moreover, this review highlights the need for both formal and informal support in caring for a sibling with MI as a way to manage the very different emotions experienced toward the brother or sister with MI. Furthermore, depending on the familial and cultural contexts, most well-siblings do not wish to assume the often-requested parent-like role.

While most recent cited international literature reports mainly negative outcomes for the well-siblings of people with MI, the review by Shivers and Textoris (2021), who primarily reviewed studies with well-siblings-as-comparison group, highlighted mixed results. Indeed, only half of the studies found that well-siblings had more negative outcomes, in terms of depressive symptoms, poorer sibling relationships and family functioning, temperament, internalizing and/or externalizing problems, or quality of life. Furthermore, authors reported that sisters had more negative outcomes than brothers, more caregiving responsibilities, and that the severity and duration of the brother/sister’s MI were related to poorer outcomes and higher caregiving rates.

Despite these evidences, the lived experience of well-siblings of people with MI is a relatively neglected and less studied topic if compared to that of being siblings of people with intellectual and developmental disabilities or chronic illness (e.g., Aikey and Goldstein, 2025; Arcous et al., 2024; Blamires et al., 2024; Cuskelly et al., 2023; Levante et al., 2025; Lummer-Aikey and Goldstein, 2020; Martinez et al., 2022; Nygård et al., 2024; Parker et al., 2020; Rea et al., 2024; Tan et al., 2025). Indeed, to the best of our knowledge, there has been no comprehensive qualitative meta-analysis specifically examining the complex and multifaceted lived experience of well-siblings of people with MI. In this vein, our qualitative meta-analysis aims to explore in depth the complex nature of this lived experience by focusing on how this affects the physical, mental, and relational health and well-being of well-siblings of people with MI, but also on their specific needs, their caregiving roles, the coping strategies they put in place to deal with their brother or sister’s MI, and the positive aspects of this experience, which are often underestimated.

## Method

In preparing this paper, we followed the American Psychological Association Journal Article Reporting Standards for Qualitative Meta-Analysis (Levitt et al., 2018) and used a qualitative meta-analytic method based upon a grounded theory approach (Charmaz, 2006; Glaser and Strauss, 1967), enabling researchers to categorize recurring experiences across various studies and then organize these categories into a hierarchical system. We used the critical constructivist grounded theory approach (CCGT; Levitt, 2021), which proved to be suitable for the present study given our interest in examining the relatively neglected topic of the lived experience of well-siblings of persons with MI, which is also constructed through socially situated meanings, as well as in how lived well-siblings’ experience develops in relation to sociocultural structures.

### Search strategy and articles collection

We conducted an electronic search in November 2025 using EBSCOHost (PsycInfo, PsycArticles, and Psychology and Behavioral Sciences Collection), EMBASE, Scopus, and the Web of Science, following the Preferred Reporting Items for Systematic Reviews and Meta-Analyses (PRISMA) guidance (Page et al., 2021). All searches included “sibling* or brother* or sister*” AND “mental ill or mental illness or mental disease* or mental disorder*” in the abstract, AND “qualitativ* or thematic analys* or grounded theory or phenomenolog* or content analys* or narrative analys*” in any field. Our review indicated that all included articles focused on well-siblings of persons with MI, which was our central inclusion criterion. For articles to be included in this study, they needed to meet the following criteria: (a) be peer-reviewed journal articles published until November 30, 2025; (b) be a qualitative study; (c) be in the English language; and (d) focus exclusively on describing the experience of well-siblings of persons with MI. The authors agreed on the inclusion or exclusion of each article based on whether it met the above inclusion criteria.

**Figure 1:**
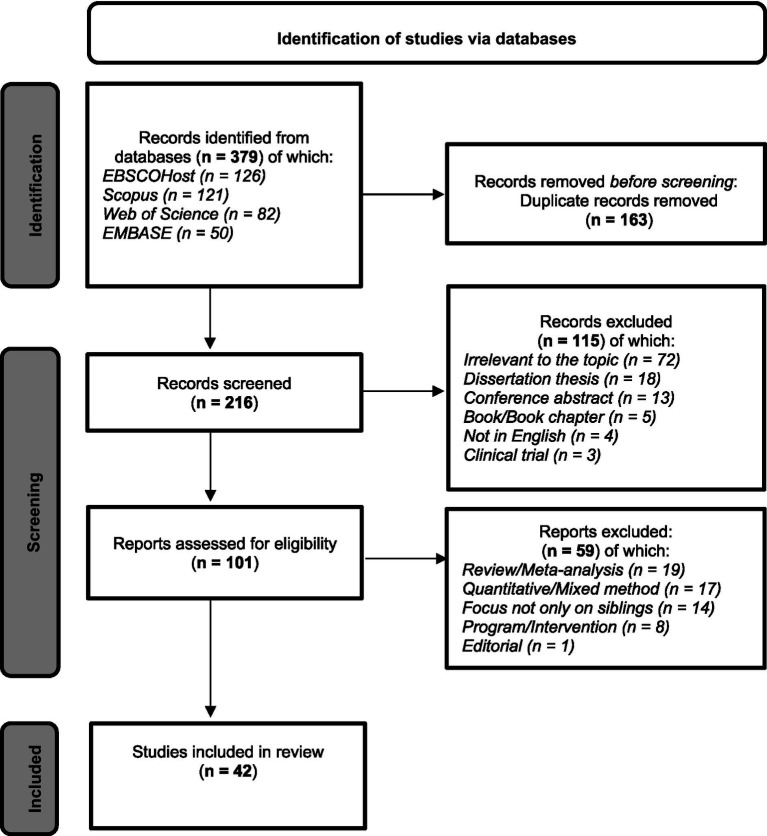
Preferred reporting items for systematic reviews and meta-analyses flow diagram. Adapted from Page et al. (2021).

A total of 379 records were identified through the electronic search. After removing duplicates, 216 records remained and were screened. Of these, 115 records were excluded (irrelevant to the topic or did not meet the inclusion criteria). A total of 101 potential papers were assessed for eligibility. Of these, 59 papers were excluded (review/meta-analysis, not specifically focused on well-siblings, quantitative/mixed method, program/intervention, editorial) (see Figure 1). Furthermore, a quality assessment was conducted for all the articles that met the inclusion criteria. The Critical Appraisal Skills Programme (CASP) Qualitative Study Checklist version 2024 (Critical Appraisal Skills Programme, 2024, Corrigan and Miller, 2004, Zauszniewski and Bekhet, 2014) was used to assess articles quality, according to which, the assessment regarded the following items: clearness in the statement of the aims of the research; appropriateness of the qualitative methodology; appropriateness of the research design; appropriateness of the recruitment strategy in addressing the aims of the research; appropriateness of data collection in addressing the research issue; adequateness in considering the relationship between researcher(s) and participants; consideration of ethical issues; sufficiently rigorous data analysis; clearness in the statement of findings; and value of the research. All items were scored according to the possible responses: “Yes,” “No,” “Cannot Tell.” All four authors were individually involved in the evaluation process, and any discrepancies were resolved in group discussions. According to the Appraisal Summary of the CASP Qualitative Study Checklist, 42 papers were judged Positive/Methodologically sound and relevant and contributed to this qualitative meta-analysis, having scored at least nine “Yes” out of 10 on the individual items (see Tables 1, 2).

**Table 1:** Characteristics of studies included in the meta-analysis.

References (country)	Qualitative method (instrument)	Well siblings (brothers; sisters) age	Mentally ill siblings (brothers; sisters) diagnosis and age
Amaresha et al. (2015) (India)	Thematic Analysis (Interviews: duration not specified)	15 (brothers = 11; sisters = 4) *M* = 33	15 (brothers = 12; sisters = 3) Schizophrenia, *M* = 30.4
Amaresha et al. (2019) (India)	Thematic Analysis (Interviews: 30–45 min.)	15 (brothers = 11; sisters = 4) *M* = 33	15 (brothers = 12; sisters = 3) Schizophrenia, *M* = 30.4
Barnable et al. (2006) (Canada)	Phenomenological Analysis (Interviews: 45–90 min.)	6 (brothers = 3; sisters = 3) 30–60	6 (brothers = 3; sisters = 3) Schizophrenia, 20–50
Brownings et al. (2023) (UK)	Phenomenological Analysis (Interviews: 57–61 min.)	8 (brothers = 4; sisters = 4) 13–20	Obsessive compulsive disorder (other data not specified)
Callio and Gustafsson (2016) (Sweden)	Content Analysis (Interviews: 40–50 min.)	5 (brothers = 3; sisters = 2) 15–20	5 (sisters = 5) Eating disorders (age not specified)
Caluza et al. (2023) (South Africa)	Phenomenological Analysis (Interviews: 37.9–55 min.)	7 (brothers = 4; sisters = 3) 22–49, *M* = 30.3	Borderline personality (other data not specified)
Dimitropoulos et al. (2009) (Canada)	Grounded Theory (Interviews: 60–90 min.)	12 (sisters = 12) *M* = 25.6	Anorexia nervosa, *M* = 25.4 (*N* not specified)
Dimmer et al. (2021) (Canada)	Thematic Analysis (Focus groups: duration not specified)	21 (brothers = 10; sisters = 11) 18–27, 20.6	Anxiety (including OCD), Bipolar disorder (*N* not specified)
Dodge and Smith (2019) (Canada)	Grounded Theory (Interviews: average 60 min.)	10 (brothers = 5; sisters = 5) 21–56, *M* = 32.2	10 (brothers = 8; sisters = 2) Schizophrenia, 26–46, *M* = 32.5
Ewertzon et al. (2012) (Sweden)	Content Analysis (Focus groups: 90–120 min.)	13 (brothers = 2; sisters = 11) 28–66, *M* = 45	13 (brothers = 6; sisters = 7) Psychosis (age not specified)
Fjermestad et al. (2020) (Norway)	Phenomenological Analysis (Interviews: not specified)	13 (brothers = 3; sisters = 10) 12–23, *M* = 15.5	10 (brothers = 1; sisters = 9) Anorexia nervosa (age not specified)
Garley and Johnson (1994) (Canada)	Phenomenological Analysis (Interviews: average 60 min.)	5 (sisters = 5) 15–18	5 (sisters = 5) Anorexia nervosa, 14–17
Gerace et al. (1993) (USA)	Content and Thematic Analysis (Interviews: average 90 min.)	14 (brothers = 3; sisters = 11) 28–45, *M* = 30	14 (brothers = 7; sisters = 7) Schizophrenia, 24–50, *M* = 33
Graves et al. (2020) (USA)	Grounded Theory (Interviews: 37–150 min.)	23 (brothers = 10; sisters = 13) 18–30	23 (brothers = 19; sisters = 4) Schizophrenia, 20–37
Graves et al. (2023) (USA)	Grounded Theory (Interviews: 37–150 min.)	23 (brothers = 10; sisters = 13) 18–30	23 (brothers = 19; sisters = 4) Schizophrenia, 20–37
Hutchison et al. (2022) (UK)	Thematic Analysis (Focus groups: average 90 min.)	14 (brothers = 6; sisters = 8) 11–19, *M =* 14.9	14 (sisters = 14) Anorexia nervosa, 13–18, *M =* 15.4
Jungbauer et al. (2016) (Germany)	Content Analysis (Interviews: 30–75 min.)	16 (brothers = 4; sisters = 12) 12–52, *M* = 24.3	16 (sisters = 16) Anorexia nervosa, 15–46, *M =* 21.9
Karlstad et al. (2021) (Norway)	Grounded Theory (Interviews iews: 28–55 min.)	10 (brothers = 3; sisters = 7) 20–31, *M =* 25.1	8 (sisters = 8) Anorexia nervosa, Bulimia nervosa, 20–30
Kitzmüller et al. (2023) (Norway)	Hermeneutic Analysis (Interviews: 80–120 min.)	2 (brothers = 1; sisters = 1) 30+	2 (brothers = 1; sisters = 1) Schizophrenia, 40+
Kovacs et al. (2019) (Israel)	Phenomenological Analysis (Interviews: 60–120 min.)	14 (brothers = 7; sisters = 7) 20–55	Schizophrenia, Bipolar disorder, Obsessive compulsive disorder, Depression, Anxiety, Eating disorder (other data not specified)
Kristoffersen and Mustard (2000) (Norway)	Hermeneutic Analysis (Interviews: average 50 min.)	16 (brothers = 10; sisters = 6) 24–61, *M* = 34	Schizophrenia (other data not specified)
Latzer et al. (2002) (Israel)	Content Analysis (Interviews: duration not specified)	9 (sisters = 9) 11–18	9 (sisters = 9) Anorexia Nervosa (age not specified)
Levkovich and Labes (2023) (Israel)	Phenomenological Analysis (Interviews: 60–90 min.)	21 (brothers = 9; sisters = 12) 18–29, *M* = 22.39	Depression, *M* = 22.41 (*N* not specified)
Liegghio (2017) (Canada)	Thematic Analysis and Grounded Theory (Interviews: 60–90 min.)	7 (brothers = 3; sisters = 4) 13–21, *M* = 15.4	7 (brothers = 6; sisters = 1) Anxiety, First-episode psychosis, Depression, Bipolar disorder, 12–22
Lindeman et al. (2023) (Norway)	Thematic Analysis (Interviews: average 120 min.)	14 (brothers = 4; sisters = 10) 23–61	14 (brothers = 13; sisters = 1) Substance use, 17–50
Lukens et al. (2002) (USA)	Grounded Theory (Focus groups: average 120 min.)	19 (brothers = 3; sisters = 16) 25–74, *M* = 43	19 (brothers = 14; sisters = 5) Schizophrenia/Schizoaffective disorder, Bipolar disorder, Depression (age not specified)
Lukens et al. (2004) (USA)	Grounded Theory (Focus groups: average 120 min.)	19 (brothers = 3; sisters = 16) 25–74, *M* = 43	19 (brothers = 14; sisters = 5) Schizophrenia/Schizoaffective disorder, Bipolar disorder, Depression (age not specified)
Main et al. (1993) (USA)	Content Analysis (Interviews: duration not specified)	11 (brothers = 3; sisters = 8) 18+	Schizophrenia (other data not specified)
McCormack and Cox (2025) (Australia)	Phenomenological Analysis (Interviews: average 60 min.)	5 (brothers = 2; sisters = 3) 40–51	5 (Brothers = 5) Bipolar disorder, Personality disorder, Substance use, Schizophrenia (age not specified)
Park and Lee (2017) (USA)	Phenomenological Analysis (Interviews: 45–60 min.)	8 (brothers = 3; sisters = 5) 20–40+, *M* = 12.7	Schizophrenia (other data not specified)
Persico et al. (2021) (France)	Phenomenological Analysis (Focus groups: duration not specified)	7 (brothers = 3; sisters = 4) 6–19,	Anorexia nervosa (other data not specified)
Riebschleger (1991) (USA)	Content analysis (Interviews: duration not specified)	20 (brothers = 9; sisters = 11) 21–65, *M* = 35	20 (brothers = 11; sisters = 3) Schizophrenia, Bipolar disorder, Organic impairment resulting in mental illness, 25–59, *M* = 29
Samuels and Chase (1979) (USA)	Content Analysis (Interviews: duration not specified)	11 (brothers = 3; sisters = 8) 24–46, *M* = 33.5	11 (brothers = 2; sisters = 9) Schizophrenia, 23–41
Schmid et al. (2009) (Germany)	Content Analysis (Interviews: average 49 min.)	37 (brothers = 13; sisters = 24) up to 30–50+, *M* = 41.6	37 (brothers = 23; sisters = 14) Schizophrenia, up to 30–50+, *M* = 39.9
Scutt et al. (2022) (UK)	Thematic Analysis (Interviews: 40–50 min.)	10 (brothers = 3; sisters = 7) 21–33, *M* = 26.7	10 (sisters = 10) Anorexia nervosa (age not specified)
Shinan-Altman and Levkovich (2024) (Israel)	Phenomenological Analysis (Interviews: average 60 min.)	25 (brothers = 11; sisters = 14) 18–29, *M* = 22.4	Depression (other data not specified)
Sin et al. (2008) (UK)	Phenomenological Analysis (Interviews: average 60 min.)	10 (brothers = 2; sisters = 8) 16–30, *M* = 22.8	9 (brothers = 8; sisters = 1) First-episode psychosis, 21–29, *M* = 24.2
Sin et al. (2012) (UK)	Phenomenological Analysis (Interviews: 30–60 min.)	31 (brothers = 9; sisters = 22) 11–35, *M* = 22.7	25 (brothers = 20; sisters = 5) First-episode psychosis, 17–36, *M* = 23.5
Stålberg et al. (2004) (Sweden)	Grounded Theory (Interviews: 45–90 min.)	16 (brothers = 8; sisters = 8) 16–55, *M* = 31	14 (brothers = 9; sisters = 5) Schizophrenia, 20–52, *M* = 32
Vukeya et al. (2022) (South Africa)	Phenomenological Analysis (Interviews: 40–60 min.)	8 (brothers = 3; sisters = 5) 23–62, *M* = 43.6	Not specified mental illness (other data not specified)
Yang et al. (2017) (Taiwan)	Content Analysis (Interviews: 60–120 min.)	10 (brothers = 3; sisters = 7) 30–65, *M* = 44.9	Schizophrenia (other data not specified)
Young and Flannigan (2021) (South Africa)	Phenomenological Analysis (Interviews: average 60 min.)	5 (brothers = 1; sisters = 4) 22–49, *M* = 29.4	Schizophrenia, Bipolar disorder (other data not specified)

**Table 2:** Results of the quality assessment through the CASP qualitative study checklist.

References (country)	Was there a clear statement of the aims of the research?	Is a qualitative methodology appropriate?	Was the research design appropriate to address the aims of the research?	Was the recruitment strategy appropriate to the aims of the research?	Was the data collected in a way that addressed the research issue?	Has the relationship between researcher and participants been adequately considered?	Have ethical issues been taken into consideration?	Was the data analysis sufficiently rigorous?	Is there a clear statement of findings?	How valuable is the research?	General appraisal
Amaresha et al. (2015) (India)	Yes	Yes	Yes	Yes	Yes	No	Yes	Yes	Yes	Yes	Positive/Methodologically sound
Amaresha et al. (2019) (India)	Yes	Yes	Yes	Yes	Yes	No	Yes	Yes	Yes	Yes	Positive/Methodologically sound
Barnable et al. (2006) (Canada)	Yes	Yes	Yes	Yes	Yes	Yes	Yes	Yes	Yes	Yes	Positive/Methodologically sound
Brownings et al. (2023) (UK)	Yes	Yes	Yes	Yes	Yes	Yes	Yes	Yes	Yes	Yes	Positive/Methodologically sound
Callio and Gustafsson (2016) (Sweden)	Yes	Yes	Yes	Yes	Yes	No	Yes	Yes	Yes	Yes	Positive/Methodologically sound
Caluza et al. (2023) (South Africa)	Yes	Yes	Yes	Yes	Yes	Yes	Yes	Yes	Yes	Yes	Positive/Methodologically sound
Dimitropoulos et al. (2009) (Canada)	Yes	Yes	Yes	Yes	Yes	No	Yes	Yes	Yes	Yes	Positive/Methodologically sound
Dimmer et al. (2021) (Canada)	Yes	Yes	Yes	Yes	Yes	Yes	Yes	Yes	Yes	Yes	Positive/Methodologically sound
Dodge and Smith (2019) (Canada)	Yes	Yes	Yes	Yes	Yes	Yes	Yes	Yes	Yes	Yes	Positive/Methodologically sound
Ewertzon et al. (2012) (Sweden)	Yes	Yes	Yes	Yes	Yes	Yes	Yes	Yes	Yes	Yes	Positive/Methodologically sound
Fjermestad et al. (2020) (Norway)	Yes	Yes	Yes	Yes	Yes	Yes	Yes	Yes	Yes	Yes	Positive/Methodologically sound
Garley and Johnson (1994) (Canada)	Yes	Yes	Yes	Yes	Yes	No	Yes	Yes	Yes	Yes	Positive/Methodologically sound
Gerace et al. (1993) (USA)	Yes	Yes	Yes	Yes	Yes	No	Yes	Yes	Yes	Yes	Positive/Methodologically sound
Graves et al. (2020) (USA)	Yes	Yes	Yes	Yes	Yes	No	Yes	Yes	Yes	Yes	Positive/Methodologically sound
Graves et al. (2023) (USA)	Yes	Yes	Yes	Yes	Yes	No	Yes	Yes	Yes	Yes	Positive/Methodologically sound
Hutchison et al. (2022) (UK)	Yes	Yes	Yes	Yes	Yes	No	Yes	Yes	Yes	Yes	Positive/Methodologically sound
Jungbauer et al. (2016) (Germany)	Yes	Yes	Yes	Yes	Yes	Yes	Yes	Yes	Yes	Yes	Positive/Methodologically sound
Karlstad et al. (2021) (Norway)	Yes	Yes	Yes	Yes	Yes	Yes	Yes	Yes	Yes	Yes	Positive/Methodologically sound
Kitzmüller et al. (2023) (Norway)	Yes	Yes	Yes	Yes	Yes	Yes	Yes	Yes	Yes	Yes	Positive/Methodologically sound
Kovacs et al. (2019) (Israel)	Yes	Yes	Yes	Yes	Yes	No	Yes	Yes	Yes	Yes	Positive/Methodologically sound
Kristoffersen and Mustard (2000) (Norway)	Yes	Yes	Yes	Yes	Yes	Yes	Yes	Yes	Yes	Yes	Positive/Methodologically sound
Latzer et al. (2002) (Israel)	Yes	Yes	Yes	Yes	Yes	No	Yes	Yes	Yes	Yes	Positive/Methodologically sound
Levkovich and Labes (2023) (Israel)	Yes	Yes	Yes	Yes	Yes	Yes	Yes	Yes	Yes	Yes	Positive/Methodologically sound
Liegghio (2017) (Canada)	Yes	Yes	Yes	Yes	Yes	No	Yes	Yes	Yes	Yes	Positive/Methodologically sound
Lindeman et al. (2023) (Norway)	Yes	Yes	Yes	Yes	Yes	Yes	Yes	Yes	Yes	Yes	Positive/Methodologically sound
Lukens et al. (2002) (USA)	Yes	Yes	Yes	Yes	Yes	No	Yes	Yes	Yes	Yes	Positive/Methodologically sound
Lukens et al. (2004) (USA)	Yes	Yes	Yes	Yes	Yes	Yes	Yes	Yes	Yes	Yes	Positive/Methodologically sound
Main et al. (1993) (USA)	Yes	Yes	Yes	Yes	Yes	No	Yes	Yes	Yes	Yes	Positive/Methodologically sound
McCormack and Cox (2025) (Australia)	Yes	Yes	Yes	Yes	Yes	Yes	Yes	Yes	Yes	Yes	Positive/Methodologically sound
Park and Lee (2017) (USA)	Yes	Yes	Yes	Yes	Yes	Yes	Yes	Yes	Yes	Yes	Positive/Methodologically sound
Persico et al. (2021) (France)	Yes	Yes	Yes	Yes	Yes	Yes	Yes	Yes	Yes	Yes	Positive/Methodologically sound
Riebschleger (1991) (USA)	Yes	Yes	Yes	Yes	Yes	Yes	Yes	Yes	Yes	Yes	Positive/Methodologically sound
Samuels and Chase (1979) (USA)	Yes	Yes	Yes	Yes	Yes	No	Yes	Yes	Yes	Yes	Positive/Methodologically sound
Schmid et al. (2009) (Germany)	Yes	Yes	Yes	Yes	Yes	Yes	Yes	Yes	Yes	Yes	Positive/Methodologically sound
Scutt et al. (2022) (UK)	Yes	Yes	Yes	Yes	Yes	Yes	Yes	Yes	Yes	Yes	Positive/Methodologically sound
Shinan-Altman and Levkovich (2024) (Israel)	Yes	Yes	Yes	Yes	Yes	Yes	Yes	Yes	Yes	Yes	Positive/Methodologically sound
Sin et al. (2008) (UK)	Yes	Yes	Yes	Yes	Yes	No	Yes	Yes	Yes	Yes	Positive/Methodologically sound
Sin et al. (2012) (UK)	Yes	Yes	Yes	Yes	Yes	No	Yes	Yes	Yes	Yes	Positive/Methodologically sound
Stålberg et al. (2004) (Sweden)	Yes	Yes	Yes	Yes	Yes	No	Yes	Yes	Yes	Yes	Positive/Methodologically sound
Vukeya et al. (2022) (South Africa)	Yes	Yes	Yes	Yes	Yes	No	Yes	Yes	Yes	Yes	Positive/Methodologically sound
Yang et al. (2017) (Taiwan)	Yes	Yes	Yes	Yes	Yes	No	Yes	Yes	Yes	Yes	Positive/Methodologically sound
Young and Flannigan (2021) (South Africa)	Yes	Yes	Yes	Yes	Yes	Yes	Yes	Yes	Yes	Yes	Positive/Methodologically sound

### Characteristics of the studies

The total number of participants across all studies was 565 (208 brothers and 357 sisters; ages ranged from 6 to 74). The number of participants in these studies ranged from 2 to 37, with a mean of 13.4 across studies. Nine studies were from the USA, 6 from Canada, 5 from the UK, 5 from Norway, 4 from Israel, 3 from Sweden, 3 from South Africa, 2 from India, 2 from Germany, 1 from Australia, 1 from France, and 1 from Taiwan. The methods used across the research articles were: phenomenological (15/42), content analysis (9/42), grounded theory (8/42), thematic analysis (6/42), and hermeneutic analysis (2/42). Two articles used a combination of content and thematic analysis (1/42) and thematic analysis and grounded theory (1/42). Most studies (36/42) used semi-structured or unstructured interviews, with reported length ranging from 30 to 159 min., while 6 studies used focus groups, with reported length ranging from 90 to 120 min. When reported, the number of siblings with MI ranged from 2 to 37, with a mean of 13.1 across studies (ages ranged from 13 to 59). Finally, the MI of the brother or sister were: schizophrenia/schizoaffective disorder (21/42), eating disorder (11/42), bipolar disorder (8/42), depression (6/42), psychosis/first episode psychosis (4/42), anxiety (3/42), obsessive compulsive behavior (3/42), substance use (2/42), borderline personality (2/42), organic impairment resulting in MI (1/42), and not specified MI (1/42) (see Table 1).

### Qualitative meta-analytic procedures

Using CCGT analytical strategies (Levitt, 2021), we conducted an in-depth examination of the results sections of each article included in the review and identified meaning units for each significant finding, thereby answering the qualitative meta-analytic research question (Giorgi, 2009). Each meaning unit was assigned a label summarizing the finding in relation to the lived experience of well-siblings of individuals with MI (e.g., “Disclosing a sibling’s MI leads to feelings of shame and embarrassment”). These meaning units were then compared to one another to form initial categories capturing common patterns in the content. These categories were subsequently compared to generate higher-order categories (clusters) reflecting shared meanings. Clusters were developed by observing and identifying commonalities and patterns across categories. The constant comparative method was applied until a central category at the top of the hierarchy of findings was identified. In developing this core category, we revisited the higher-order categories that had emerged through the constant comparison process within the hierarchy and considered how best to capture the lived experience of well-siblings of individuals with MI in a concise and meaningful way.

### Authors’ positionality and reflexivity

The first author is a White, cisgender man, clinical psychology researcher and psychoanalyst, born in Italy, and lost a brother to a rare autoimmune syndrome. The second author is a White, cisgender woman, clinical psychology researcher, and training psychoanalyst, with no experience of siblings with mental or physical illness. The third author is a White, cisgender man, a developmental psychology researcher and training psychotherapist, with no experience of siblings with mental or physical illness. The fourth author is a White, cisgender woman, developmental psychology researcher and psychotherapist, with the experience of two daughters, one of whom suffers from a chronic illness. The perspectives of some of the researchers (specifically the first and fourth authors) who participated in this study were strongly influenced by their reading and engagement with the literature on the lived experiences of well-siblings of people with MI, their previous clinical experiences with sibling clients, as well as their personal family experiences with chronic (non-mental) illness. Throughout the research process, they continued to engage in reflective discussions about their perspectives and expectations regarding the topics under study and to present the results faithfully, facilitated by constant exchange and discussion with the other two researchers, who were not personally involved in the experience of chronic illness. The four researchers worked together to minimize the undue influence of the first and fourth authors’ positionality and subjective experiences through memoing, discussions, and self-reflection. All authors collected and reviewed the published studies together and wrote the article.

### Methodological integrity checks

Among the wide range of procedures available to strengthen a study’s methodological integrity (Levitt et al., 2017), we selected three checks: memoing, establishing consensus between the investigator and supervisor during both data selection and analysis, and monitoring saturation. Memoing was used to minimize the influence of the investigators’ own perspectives on the analytic process and to consider ways of adapting methods to ensure that the analysis remained grounded in the data. It also served as a tool for documenting theoretical reflections, methodological justifications, and coding notes. NVivo version 15 (Lumivero, 2025) was used to this aim. To foster open and honest dialogue about the data and emerging findings, we adopted a critical-constructivist approach to consensus (Levitt et al., 2021), which values the researchers’ diverse lived experiences, positionalities, and knowledge bases. This approach encouraged acknowledgment of differences among the researchers themselves (e.g., power dynamics, areas of expertise, personal experiences) and between researchers and participants (especially in terms of lived experience). We reflected carefully on how these differences might shape the analysis and took deliberate steps to consider interpretations that may not have emerged from our initial readings of the data. In grounded theory methodology, monitoring saturation enhances researchers’ confidence in the comprehensiveness of the analysis by indicating when additional data no longer contribute to the development of new categories or variations within them. In this study, saturation was reached at the 38th study out of the 42 included. Moreover, no discrepancies were found between the assessment of study quality conducted using CASP Qualitative Study Checklist version 2024 and Levitt and colleagues’ methodological integrity framework (Levitt et al., 2017; Levitt et al., 2021).

## Results

The hierarchy of the qualitative meta-analysis consisted of five clusters (see Table 3). At the top of this hierarchy was the core category, which included five clusters comprising 17 categories, themselves made up of 45 subcategories and a total of 485 coded meaning units. In the sections that follow, we describe the clusters, the categories, and the core category to highlight the main findings. These numbers should not be interpreted as estimates of the percentage of agreement across the 42 included studies, as each study explored different aspects of well-siblings’ lived experiences of having a brother or sister with MI. Rather, they indicate how many studies found each idea to be salient for well-sibling participants.

**Table 3:** Clusters, category titles, and the number of studies that contributed meaning units to each.

Cluster and categories (number that contributed out of 42)
Cluster 1: MI significantly affects well-siblings’ emotions, health, and well-being (*N* = 40)
- Category 1.1: Wide range of emotions towards the brother or sister with MI (*N* = 30)
- Category 1.2: Wide range of emotions/preoccupations towards oneself (*N* = 27)
- Category 1.3: Wide range of emotions towards the outside world (*N* = 20)
- Category 1.4: Burden and impact on health and well-being (*N* = 25)
- Category 1.5: Impact on academic, working, and personal life (*N* = 22)
Cluster 2: Well-siblings feel excluded, abandoned, or treated as secondary (*N* = 26)
- Category 2.1: Having to grow up quickly and parentification (*N* = 16)
- Category 2.2: Being treated as secondary (*N* = 16)
- Category 2.3: Self-marginalization (*N* = 13)
Cluster 3: Well-siblings express their need for information and support (*N* = 29)
- Category 3.1: Need for information on MI (*N* = 23)
- Category 3.2: Need for support from significant others (*N* = 14)
- Category 3.3: Need for support from others having the same lived experience (*N* = 10)
- Category 3.4: Need for support from mental health practitioners (*N* = 25)
Cluster 4: Well-siblings also experienced positive changes and personal growth (*N* = 27)
- Category 4.1: Positive changes in sibling and family relationships (*N* = 24)
- Category 4.2: Personal growth (*N* = 19)
- Category 4.3: Hope in the future (*N* = 8)
Cluster 5: Well-siblings adopt different caregiving roles and coping strategies (*N* = 34)
- Category 5.1: Different caregiving roles (*N* = 24)
- Category 5.2: Different coping strategies (*N* = 27)
Core category: Being the brother or sister of someone with a MI is a difficult experience, involving a strong impact and a need for information and support, but also personal and relational growth (*N* = 42)

### Cluster 1: MI significantly affects well-siblings’ emotions, health, and well-being

The first cluster contained descriptions of siblings’ recognition of the impact of their brother or sister’s MI on their emotional, academic, working, and personal lives. Forty articles contributed to the cluster, and the 6 categories that make up the cluster are described in more detail below.

#### Category 1.1: Wide range of emotions towards the brother or sister with MI

Well-siblings across 30 studies described a wide range of emotions toward the brother or sister with MI, varying from anger, resentment, and blame to closeness, admiration, and pity. In this vein, a sister said: “I was angry with her for trying to kill herself, leaving us behind…” (Caluza et al., 2023, p. 4). Another sister affirmed: “[I feel] resentful … definitely. Very angry. It’s difficult to be angry with anorexia and not [sister]” (Hutchison et al., 2022, p. 4). One sibling said: “My family has been suffering because of M [the patient]‘s illness. I am not saying that it is her fault. But its impact on us was quite significant” (Park and Lee, 2017, p. 100). Another one, affirmed movingly: “There’s nothing like a sibling relationship and I mean, they’re the people that teach you, and they teach you a lot of things about life, even if it’s screwed up and a bit crazy” (McCormack and Cox, 2025, p. 13). Siblings also expressed a sense of loss and mourning for the brother or sister they knew before the onset of MI. For example, talking about her anorexic sister stated: “And she became kind of, in the illness, she was not herself. She was a completely different person” (Fjermestad et al., 2020, p. 5). Another participant said: “Yes, you know when someone dies they die. In that situation you have a reason to be sorry at times, but you can obviously go back and think about the lost one. But here you have lost someone, but you have not lost them. Here you don’t have permission to grieve for her in the same way” (Kristoffersen and Mustard, 2000, p. 25). Similarly, a brother affirmed: “I think that is another hard thing to do, to accept who she is now and is different from who she was, and she will never be the same and try to go on” (Dodge and Smith, 2019, p. 41). It seems that well-siblings’ affective life is occupied by an attempt to comprehend and master feelings experienced toward their brother or sister with MI.

#### Category 1.2: Wide range of emotions/preoccupations towards oneself

Well-siblings across 27 studies mainly expressed negative emotions towards themselves due to constant contact with their brother or sister’s MI, particularly guilt. In this vein, a sister affirmed: “I definitely think that anger is a difficult emotion to process, especially because I can feel very guilty over anger, like the anger that I felt towards my sister, I still feel very, very guilty over that and I haven’t forgiven myself for the way that I treated her” (Scutt et al., 2022, p. 7). Similarly, a brother said: “I’ll never lose that guilt, if anything I try to use it as a motivator… It’s always going to be there, it’s never going to disappear, I’ve just got to use it… That’s a powerful weapon” (McCormack and Cox, 2025, p. 15). But the well-siblings also describe their experience of powerlessness in being unable to help either their brother or sister with MI, or the entire family, or in fearing a possible ‘contagion’ by MI. In this vein, a sister reported: “I’ve had dreams where I was schizophrenic. People were dragging me away and I was trying to explain things, but all that came out of my mouth was screaming” (Lukens et al., 2004, p. 493). Furthermore, well-siblings also frequently reported the fear of becoming mentally ill or transmitting the MI to their children, as a participant said: “I don’t know if the diagnosis had been made then, but it was tough when he was hospitalized. I felt a strong concern about getting ill myself and I was fairly paranoid about it” (Stålberg et al., 2004, p. 452). And a sister stated: “I’m thinking about having a baby. Do I adopt? Because what would I do if I had a mentally ill daughter?” (Lukens et al., 2004, p. 493).

#### Category 1.3: Wide range of emotions towards the outside world

Well-siblings across 20 studies described their different emotions toward the outside world, mainly shame and embarrassment. A brother explicitly said that “it was embarrassing and uncomfortable talking to people about his condition” (Amaresha et al., 2019, p. 417). Well-siblings also have to deal with stigma towards MI, as said by a participant: “I think the hardest thing is coping with it [schizophrenia] initially… It’s a mystery to people and there is such a stigma still attached to it that people are just totally bewildered” (Gerace et al., 1993, p. 641). Similarly, another sibling stated: “Some might be ashamed of it and thus don’t dare do anything because of the fear it will come out in the open and that you will be labeled” (Stålberg et al., 2004, p. 449). These emotions seem to be reinforced in situations where the family itself, particularly the parents, finds it difficult to talk openly about a child’s MI. A brother reported: “They always told me not to tell anyone. I remember that my mother insisted that I keep this a secret, and also my father” (Levkovich and Labes, 2023, p. 8). Similarly, a sister said: “My parents were like, “You cannot tell people about [brother] … “You don’t want this kind of thing getting around.”… I just never wanted to have friends over… There’s no explanation I can give because even if I felt comfortable enough to be like, “He’s bipolar,” I wasn’t supposed to do that… It’s hard not being totally honest with your friends… it was suddenly, “No, we can’t go to my house.”” (Dimmer et al., 2021, p. 7).

#### Category 1.4: Burden and impact on health and well-being

Well-siblings across 25 studies mainly described their feelings of burden, in terms of sorrow, regret, anxiety, stress, frustration, and depression, among others, as well as the impact of their brother or sister’s MI on their health and well-being. A sister affirmed: “It stresses me out a lot… If I could choose to be anywhere, it would be anywhere but home, because home, there’s always problems, or there’d just be issues with my brother and then I could hear all about it and then I have to get involved” (Liegghio, 2017, p. 1240). Another well-sibling explicitly said: “I have a horrible life because I have to give all my strength to dealing with my sister or with the situation” (Ewertzon et al., 2012, p. 160). Economic burden also characterizes the lived experience of well-siblings of a brother or sister with MI, as a brother said: “I have both a home loan and a car loan. I work in a factory that makes eyeglasses, so it’s not like I have millions and millions. I have no idea how to pay the hospitalization fee now… It’s a long way to go, a bottomless pit” (Yang et al., 2017, p. 5). The economic burden adds to the emotional burden, leaving the well-siblings feeling overwhelmed, especially when the sibling with MI loses his income because of the MI, as a sister said: “I was very worried because she was very helpful at home, she used to buy furniture, buy clothes even for me you see. Because my mother didn’t get paid much… So she was a breadwinner, I can say that. So I felt very sad, very sad because I knew that I would struggle this much” (Young and Flannigan, 2021, p. 3). Talking about the impact on her health, a sister affirmed: “You know I didn’t mind helping, but I do think the stress again played a big part in later mental health issues” (Scutt et al., 2022, p. 6). The impact on the mental and physical health of well-siblings seems to be particularly significant in the brothers and sisters of individuals with eating disorders. Indeed, in these situations, family life is particularly disrupted, especially during family activities such as mealtimes or holidays, and well-siblings are often required to take on additional responsibility for monitoring the health of their brother or sister with MI, as a participant said: “Before she was like my equal… Now she’s like a little puppy, you know. I feel like I have to carry her around with me, like make sure she’s OK” (Garley and Johnson, 1994, p. 161). But also well-siblings of people affected by other MI experience challenging situations, having a substantial impact on their health and well-being, as stated by a brother, “It was like we were in a nightmare, we couldn’t sleep at night and during the day we had to watch her” (Caluza et al., 2023, p. 4).

#### Category 1.5: Impact on academic, working, and personal life

Well-siblings across 22 studies described the impact of their brother or sister’s MI on several domains of their lives. For example, a brother affirmed: “I am having lot of pressure both personally and professionally. I feel restless… Not able to spend time with my fiancée and friends. Mother calls me and complains about my sister. All these things disturb me a lot” (Amaresha et al., 2015, pp. 20–21). Similarly, another well-sibling said: “I think about her [sister with schizophrenia] a lot… Sometimes it affects my work and I have a hard time concentrating. When studying, I tend to think about [sibling] and have a hard time getting through it” (Gerace et al., 1993, p. 643). The well-siblings clearly describe the enormous difficulty of balancing the need to care for their brother or sister with MI, with the family as a whole, and with their own relationships and romantic lives, feeling as if they always have to put their own lives on the back burner. In this vein, a sister said: “I wasn’t able to live my own life because there was always someone I felt guilty about or needed to help or offer support to” (Lindeman et al., 2023, p. 7). Similarly, another well-sibling expressed his emotional conflict: “Do I stop my life to come back and help take care of her or keep going on with my life, and that leaves you pretty guilty. She wants you there to help her but you have your own life you can’t stop it” (Dimitropoulos et al., 2009, p. 358).

### Cluster 2: Well-siblings feel excluded, abandoned, or treated as secondary

The second cluster contained descriptions of well-siblings’ feelings of exclusion, abandonment, or being treated as secondary, but also of having to keep their needs or feelings to themselves or suppress them to avoid troubling or burdening others, mainly parents. Twenty-six articles contributed to the cluster, and the 3 categories that make up the cluster are described below.

#### Category 2.1: Having to grow up quickly and parentification

Well-siblings across 16 studies described having to grow up quickly because of their brother or sister’s MI. A sibling affirmed: “I had to be an adult even though I was still a teenager… The hardest part of it was having to grow up so quickly” (Dimmer et al., 2021, p. 5). Similarly, a sister affirmed: “I think my sister’s illness made me grow up too quickly. I was exposed to the world of mental health at a relatively young age” (Shinan-Altman and Levkovich, 2024, p. 2664). Well-siblings also described feeling pressured to take on a parental-like role, while “the patient acts as the “baby”” (Latzer et al., 2002, p. 277). For example, a sister said: “I wouldn’t say I parented as such, but I definitely felt very responsible, felt very guilty, I felt like it was my job to help and stuff” (Scutt et al., 2022, p. 6). Similarly, another sister said: “On the weekends I would come home from university and spend lots of time there doing things my mother never had time for, like cleaning the house, taking the dog for a walk, doing laundry, anything that my mother could never find time for because she was busy with my sister” (Levkovich and Labes, 2023, p. 8). This premature growth, together with parentification, makes the lived experience of well-siblings of people with MI particularly difficult, especially given the increasingly pressing responsibilities they feel they must take on. As a sibling of an anorexic girl said: “Her and my father do not see eye to eye at all. He has a temper. I’m the only one there that can really protect her” (Dimitropoulos et al., 2009, p. 354).

#### Category 2.2: Being treated as secondary

Well-siblings across 16 studies described feeling treated as secondary. For example, a sister affirmed: “It made my parents miss out on a lot of important moments in my life. They missed my high school graduation because she was in the hospital. They just didn’t notice when things were wrong with me because they were constantly taking care of her” (Dimmer et al., 2021, p. 8). Another well-sibling stated, “Why doesn’t he or she snap out of it? Why do I need to be involved? I’m not a parent. My sibling is getting all the attention. What about me?” (Riebschleger, 1991, p. 97). Similarly, a brother affirmed: “My parents had to focus all of their attention on my brother” (Graves et al., 2023, p. 11). It is as if the well-siblings have learned that turning to their parents or relatives is ineffective because their emotional resources are almost exclusively devoted to their child with MI, and this could lead well-siblings to feel neglected and put aside, as stated by a participant: “So when aunties and such ask; How is your sister? Your sister, your sister… I just noticed that, now, what about me? I always became so frustrated. I always became like: Do I exist in this world, really, or is it just her?” (Fjermestad et al., 2020, p. 8).

#### Category 2.3: Self-marginalization

Well-siblings across 13 studies described feeling that there is no real place for them in the family, that they must sacrifice themselves and push themselves to the back, because the needs and requirements of their brother or sister with MI always come before theirs. A sister affirmed: “I think it’s always their needs above yours… Not that your parents [are], like, being intentionally, like, neglectful of you, but… I always understood that, that that’s how it needed to be” (Hutchison et al., 2022, p. 5). Similarly, a brother said: “If I’m experiencing problems or feeling depressed… It’s like I wouldn’t want to put that on my parents since they’re already dealing with that stuff so much” (Dimmer et al., 2021, p. 9). Often, the greatest fear is placing an additional burden on parents who are already overwhelmed by the MI of their other child, which can lead to loneliness. As a brother stated: “It’s like being unable to feel anything that brings happiness… You don’t see anyone anymore, you shut yourself up, inside your own bubble… As if you were truly alone” (Persico et al., 2021, p. 5). This difficulty in expressing emotions, needs, and feelings can be explained by a reluctance to ask for attention from their already-overburdened parents, and also by the fact that when they look at their ill sibling’s needs, theirs seem less and less important.

### Cluster 3: Well-siblings express their need for information and support

The third cluster contained descriptions of well-siblings expressing their need for information on MI issues, as well as their need for support from significant others and from mental health practitioners. Twenty-nine articles contributed to the cluster, and 4 categories making up the cluster are described.

#### Category 3.1: Need for information on MI issues

Well-siblings across 23 studies described their need for information on MI. For example, a brother said: “We had to find out on the internet what borderline personality disorder after the doctors told us her diagnosis. I still think it would help us as a family if they explained this illness in more details” (Caluza et al., 2023, p. 5). Another well-sibling expressed his disappointment in not having received information: “When the family would call for information, they were told they would have to check with the patient before they would give out information. And, of course, when they checked with an acutely psychotic patient, she refused the family to have the information” (Main et al., 1993, p. 149). Similarly, a well-sibling affirmed: “It was frustrating because we didn’t know how to deal with what was happening to him. We didn’t know at that point that it was schizophrenia… It was frustrating because we were afraid of the unknown” (Barnable et al., 2006, p. 252). Indeed, most well-siblings of people with MI, especially in the early stages, experience the drama of not knowing what it is, how to react, or how to help, as a sibling said: “You can’t get information and you don’t know how to act. I understand concerns about confidentiality and civil liberties, but you’re kind of operating in a vacuum” (Lukens et al., 2002, p. 359).

#### Category 3.2: Need for support from significant others

Well-siblings across 14 studies described their need for support from significant others. For example, a brother said: “I was lucky to have competent friends to talk to. Especially one friend who studied psychology supported me” (Kitzmüller et al., 2023, p. 5). Similarly, another well-sibling affirmed: “I would discuss with my second brother when I have difficulty (caring for our sister with schizophrenia) and unable to manage. Although his financial condition is not so good, he would find some time to visit our ill sister” (Yang et al., 2017, p. 5). The role of friends, other well-siblings, and the nuclear or extended family is particularly significant in alleviating the burden experienced by well-siblings of people with MI. Well-siblings also identified parental advice as an essential source of support, as stated by a sister: “I’d have to say my mom’s a big one. I’ve seen her walk through horrible things and she’s got a beautiful strength about her and she’s just very wise and so I definitely, my parents are big ones” (Graves et al., 2023, p. 9).

#### Category 3.3: Need for support from others having the same lived experience

Well-siblings across 10 studies described their need for support from others who have the same lived experience of siblings’ MI. A brother said: “If there is an opportunity, I would be interested to meet other families who have patients with similar illnesses. I want to understand their struggles and learn how they are caring for their relatives” (Amaresha et al., 2015, p. 20). Describing his experience in participating in a sibling group, a brother affirmed: “at first we thought we were the only ones. But as soon as we were here… We felt less lonely. We were able to talk about things” (Persico et al., 2021, p. 6). Furthermore, interacting with brothers and sisters who have had or are going through the same experience allows well-siblings to express themselves without being disturbed by the judgments of other people who have no experience of MI, but also learning by their experience, as a well-sibling said: “In the sibling group, I was just amazed and so happy that there were people out there like us, people who had this problem and were dealing with it in a different way” (Lukens et al., 2002, p. 360).

#### Category 3.4: Need for support from mental health practitioners

Well-siblings across 25 studies described their need for specialistic support. For example, a well-sibling said: “They need some guidance on how to deal with day to day situations that arise. I think it’s very important that they (physicians and nurses) don’t alienate the family, that they let the family feel that it is okay to call and ask questions” (Main et al., 1993, p. 150). Similarly, another well-sibling affirmed: “If maybe there was psychoeducation about mental illness in our community maybe there will be an understanding and even us we will know what to do when we face these challenges” (Vukeya et al., 2022, p. 7). The well-siblings clearly describe how crucial the supportive role of mental health practitioners is to them. However, these practitioners sometimes seem to blame the families, thereby creating an additional emotional burden to deal with, or not supporting them, as a well-sibling said, talking about her sister’s difficulty in getting help and feeling blamed for not being able to convince her: “It was difficult to get help for him. That was frustrating. He wouldn’t go along with our suggestions and see a doctor. We were frustrated knowing there was a problem and not being able to get the professional help he needed” (Barnable et al., 2006, p. 255).

### Cluster 4: Well-siblings also experienced positive changes and personal growth

Despite the significant challenges of the lived experiences of well-siblings of people with MI, the fourth cluster contained descriptions of well-siblings experiencing positive changes and personal growth at different levels in the face of MI, as well as hope in the future. Twenty-seven articles contributed to the cluster, and the 3 categories that make up the cluster are described below.

#### Category 4.1: Positive changes in sibling and family relationships

Well-siblings across 24 studies described positive changes in both sibling and family relationships. Talking about his sibling relationship, a sister said: “I believe that this may have led us to take greater care of each other. I think we are more aware of each other… I suppose we have become closer, we do more things together, previously we did nothing” (Callio and Gustafsson, 2016, p. 617). Similarly, another sister affirmed: “We became closer. She depends on me for any help; she’s always welcome here… It’s brought us much closer than before” (Sin et al., 2008, p. 37). It seems that for some well-siblings, the experience of a brother or sister’s MI has been an opportunity for a positive change in their relationship that would not otherwise have happened. A sister said: “We first became best friends when she was anorexic. We really grew closer because of this” (Jungbauer et al., 2016, p. 81). In some cases, family relationships have also improved significantly. For example, in very closed families with poor communication, well-siblings perceive MI as an opportunity for greater closeness and openness to dialogue, as stated by a well-sibling: “It has affected… [the family] but in a way, like, we’ve become a really, really close family as well. Very close, so, at the same time, it’s a good experience, in getting us all close” (Sin et al., 2012, p. 56).

#### Category 4.2: Personal growth

Well-siblings across 19 studies described their personal growth in contact with their brother or sister’s MI. For example, a sister said: “I’ve matured a lot and become more sensitive to my surroundings. I understand that I have strength in this area” (Shinan-Altman and Levkovich, 2024, p. 2666). Another participant clearly stated that siblings’ MI “has some good effect as well because it’s made me…open up to how people feel and be more aware” (Sin et al., 2008, p. 37). Over the years, the well-siblings developed a new perspective, stemming from their unique lived experience, which leads them to feel a strong sense of understanding, compassion, and kindness toward people struggling with mental health issues, as well as a better sense of themselves. In this vein, a well-sibling stated: “I mean it hasn’t been positive at all… Perhaps it has made me more compassionate”, as another sibling: “I can really tune in with people’s pain-this is both a curse and a blessing. I’ve developed a deep compassion for people and [learned] not to make quick judgments” (Lukens et al., 2004, p. 494). Another well-sibling talked about “development of particular competences as a result of caring for ill sibling” (Schmid et al., 2009, p. 331), with particular reference to empathy, responsibility towards weaker people, consideration, tolerance, flexibility, and self-confidence.

#### Category 4.3: Hope in the future

Well-siblings across eight studies described their hope in the future derived from the contact with their brother or sister’s MI. For example, a well-sibling said: “I hope that he (patient) can get married and have children. He will make such a great father. When we go to the grocery shopping together… He chooses the best fruit. He is very good with my children too. He is a great uncle. I wish he meet a girl who can understand his illness” (Park and Lee, 2017, p. 100). Indeed, becoming familiar with MI, understanding its causes, and learning how to cope with it helps develop new perspectives on the life of a brother or sister with MI and fosters feelings of hope, as stated by a well-sibling: “Yes, I guess I thought she would get well. That was my motto and it still is… I think everyone should have that motto, even though for me it has been perhaps 8 years that I have had that motto, still it has helped me a lot” (Fjermestad et al., 2020, p. 11).

### Cluster 5: Well-siblings adopt different caregiving roles and coping strategies

The fifth cluster contained descriptions of well-siblings’ different caregiving roles and various coping strategies in dealing with their brother or sister’s MI. Thirty-four articles contributed to the cluster, and 2 categories making up the cluster are described.

#### Category 5.1: Different caregiving roles

Well-siblings across 24 studies used a variety of ways to assume and carry out the caregiving role for their brother or sister with MI. One of these ways, for example, was serving as information relayer for the family: “My parents would certainly rather I talked with doctors because they feel I understand things better than they do… I always feel like I’m trying to explain things to my family and mediate things” (Main et al., 1993, pp. 151–152). But well-siblings described very different approaches in adopting their caregiving role, going from planning care, having an active engagement with mental health professionals, and including the ill sibling in their social life, as an older brother stated: “I think part of it is now trying to get him happy and stuff. And I’ve been taking him to social activities and taking him to the gym and spending extra time with him. It helps” (Sin et al., 2012, p. 55). Other reported caregiving roles were helping the sibling with MI or other family members in navigating crises, as a brother said: “I would say my mom is definitely the one that takes care of tasks and that sort of thing… I would be the emotional support for my mom as well as [for my brother]. If I’m around during my brother’s outburst, I tend to go and talk to him and separate him from whatever the situation is” (Graves et al., 2020, p. 10). Well-siblings also reported assuming active and concrete caregiving roles for the brother or sister with MI, as said by a sister: “I was totally there. I was fully engrossed, in action mode. I didn’t ask too many questions. Apparently I understood there really wasn’t anyone to talk to. It was an emergency situation… I often sat beside her all night long, I engaged in guided imagery, lots of discussions, I was totally drawn in and devoted myself totally, and afterwards I was totally drained” (Levkovich and Labes, 2023, p. 7). Similarly, a sister said: “I make sure he takes his medication, gets to sleep on time and I wake him up for his appointments, even if he does not do these things I have tried my best” (Sin et al., 2012, p. 55).

#### Category 5.2: Different coping strategies

Well-siblings across 27 studies described their different coping strategies in facing their brother or sister’s MI, going from suppression, normalization, avoidance, blame, isolation, and projection, to acceptance. Another kind of reported coping technique was omnipotence, as clearly stated by a brother: “I had this fantasy my omnipotent presence would change the situation. But once again the Doctor said he needed to be hospitalized, and I said, ‘He’s the Doctor.’ So I became instrumental in the whole thing…” (Samuels and Chase, 1979, p. 31). However, one of the most widely described coping strategies is undoubtedly distancing, as expressed by a sister: “She has been ill for so many years that you feel you have to distance yourself a little, or you are going to go crazy yourself” (Karlstad et al., 2021, p. 5). Another well-sibling affirmed: “Yeah I think I just needed to just like escape for a few minutes” (Brownings et al., 2023, p. 470). Similarly, a sister sated: “I need a certain distance. It’s kind of my distancing from the situation with all that, that I am the closest to her and talk to her the most, and the most included, I still took quite a distance for myself, to protect myself and my family, and my sanity” (Kovacs et al., 2019, p. 1188). In this vein, well-siblings affirm the necessity of maintaining a balanced distance to protect their freedom and their own lives. The necessity to distance oneself from the brother or sister with MI, to disconnect to “recharge the batteries”, is also described as strictly connected with the need to reconnect with one’s own life, as explicitly another well-sibling said: “I think keeping somewhat of a distance from it, don’t deny it, just somewhat of a distance. Living fully in your own life… Keeping somewhat of a distance, staying busy in your own life, and if it means having a specific hobby that’s great” (Dimitropoulos et al., 2009, p. 358).

*Core Category: Being the Brother or Sister of Someone with a MI is a Difficult Experience, Involving a Strong Impact and a Need for Information and Support, but Also Personal and Relational Growth.*

Although the lived experience of well-siblings of people with MI varies depending on the specific pathology of their brother or sister, as well as on their cultural contexts, the 42 articles included in the meta-analysis revealed some significant patterns and meanings. The results showed that participants needed to describe how profound was the impact of their brother or sister’s MI on their own mental health and well-being and on several aspects of their own’s life. The results also showed the need for well-siblings to talk about the different and complex emotions regarding their having to grow up quickly, as well as about how they felt in their families, overwhelmed by their brother or sister’s MI (see Cluster 1 and 2). Well-sibling participants also felt the necessity to talk about their needs for information about MI and support in facing it as a significant help to adapt to and carry on the difficult role of caregiver and trying to find the best coping strategies (see Clusters 3 and 5). Finally, despite the significant challenges of their lived experience of having a brother or a sister with MI, well-siblings clearly talked about positive changes in sibling and family relationships, of opportunities for personal growth, as well as of new confidence and hope in the future (see Cluster 4). The core category demonstrated the relationship between the clusters and articulated the main meanings, challenges, and resources of the lived experience of well-siblings of people with MI. In particular, it highlighted three elements that strongly characterize it: the *strong emotional impact*, with all its facets; the *need for support*, both individually and within the family; and the positive influence in terms of *personal and relational growth*.

## Discussion

The findings of the present qualitative meta-analytic study synthesize the qualitative findings that have resulted from the research literature examining the lived experience of well-siblings of people affected by MI and provide robust evidence-based responses to it. As previously mentioned, al the 42 studies included in the review demonstrated good levels of quality and validity according to the Critical Appraisal Skills Programme (CASP) Qualitative Study Checklist, version 2024, being evaluated Positive/Methodologically sound.

Firstly, our results depicted robust findings from the qualitatively meta-analyzed studies, in line with the international literature on the topic, confirming the varied nature of the emotions that affect siblings’ relationships with their brother or sister with MI, ranging from worry, anger, shame, and frustration to closeness, pity, and pride. Indeed, it emerges that well-siblings feel very mixed and conflicting emotions that are part of navigating their complex family roles. However, among the most frequently expressed emotions about oneself are guilt for not doing enough for a brother or sister with MI, together with helplessness (Barak and Solomon, 2005; Heneghan et al., 2024; Lively et al., 1995). Another significant result is that well-siblings experience grief over the “loss” of the sibling they knew before the MI onset. Indeed, facing the trauma the MI provoke in transforming the relationship with the brother or sister with MI, that would never be the same, well-siblings have to accept the sever and/or chronic condition of the MI, as well as adjust to illness-related personality and behavioral changes of their brother or sister with MI, which implies a mourning process (Kinsella et al., 1996; Leith and Stein, 2012; Leith et al., 2018; Lively et al., 1995).

Moreover, as several studies have highlighted, our findings also highlight that major themes emerging in well-sibling caregivers’ experience in facing the outside world are embarrassment, stigma, and social exclusion related to their brother or sister with MI. Indeed, the well-siblings of people with MI often face complex emotional and social situations characterized by shame, embarrassment, stigma, and different forms of social exclusion. These feelings can stem from reactions to the outside world, such as prejudice, avoidance, or judgment from peers, healthcare personnel, and others, as well as from internalized beliefs influenced by cultural narratives surrounding MI. Shame and embarrassment can lead well-siblings to hide their family situation, limit social interactions, or avoid discussing their brother or sister’s condition, thereby reinforcing isolation. Stigma, whether public or internalized, can undermine their sense of identity, causing them to question their own psychological vulnerability or social value. As a result, many well-siblings experience various forms of social exclusion, feeling themselves misunderstood, unsupported, or “different” from peers (Corrigan et al., 2006; Dijkxhoorn et al., 2023; Hovland et al., 2025; Tharoor and Ganesh, 2015; van der Sanden et al., 2015; van Zelst et al., 2015; Wulandari et al., 2024). However, as we will discuss further, these experiences can also foster resilience, empathy, and a greater capacity for responsibility, albeit at the cost of emotional burden that is often unrecognized.

Furthermore, the findings from the present qualitative meta-analysis provided evidence from 40 studies on the emotional and economic burden experienced by well-siblings, as well as on the impact of well-siblings’ caregiving on their own health and well-being and on several domains of their lives. Indeed, according to literature findings, the well-siblings of people with MI are more likely to report symptoms of anxiety, stress, and depression compared to individuals without a brother or sister with MI. Literature findings, also confirm our results, thus indicating that living with a brother or sister with MI is emotionally demanding because well-siblings may witness difficult behaviors, mood swings, crisis situations, and experience more conflicting family relations, which can lead to ongoing stress and worry (Brolin et al., 2024; Chen and Lukens, 2011; Hatfield and Lefley, 2005; Jayasinghe et al., 2023b; Krzeczkowski et al., 2022; Lively et al., 1995; Ma et al., 2020; Ólafsdóttir et al., 2020; Pierazzuoli et al., 2020; Shivers et al., 2023; Wolfe et al., 2014). The results also highlighted that the well-siblings of people with MI often experience a profound and pervasive conflict between academic and/or work life and the need to care for their brother or sister with MI, with significant difficulty maintaining a balance between the needs of their sibling with MI, their own relational and family life, and their own needs (Barak and Solomon, 2005; Friedrich et al., 2008; Jayasinghe et al., 2023b; Nygård et al., 2024; Smith and Greenberg, 2008). Moreover, the results provided evidence of the significant financial burden, which, in turn, increases the emotional burden of the well-siblings of a brother or sister with MI (Alfanta et al., 2024; Barak and Solomon, 2005; Clark and Drake, 1994; Chen and Lukens, 2011; Greenberg et al., 1999; Ólafsdóttir et al., 2020; Sanders et al., 2014; Shivers et al., 2023).

The findings from the present qualitative meta-analysis provided evidence from 26 studies (see Cluster 2) that well-siblings of people with MI often feel forced to grow up prematurely, taking on parental-like roles more than those of a peer. Facing unpredictable symptoms or crises, they may become caregivers, emotional regulators, or mediators within the family, taking on responsibilities far beyond their developmental stage. At the same time, many well-siblings deliberately marginalize themselves within the family system in an effort to avoid adding to the already overwhelming burden on their parents. They may suppress their own needs, minimize their emotions, or strive to appear “problem-free,” believing that any additional demands would be unfair to a family already strained by the difficulties of their child with MI. This dynamic often leads well-siblings to feel secondary or invisible, as the parents’ attention and resources are understandably directed toward the child who requires more intensive care. Over time, such experiences can shape their sense of identity and interpersonal patterns, leaving a lasting impact that often goes unrecognized or insufficiently supported (Brolin et al., 2024; Cui et al., 2025; Heneghan et al., 2024; Jayasinghe et al., 2023a, 2023b; Kinsella et al., 1996; Lecciso et al., 2025; Levante et al., 2023; Lukens et al., 2004; Pierazzuoli et al., 2020; Sanders et al., 2014; Sommantico et al., 2020).

Additionally, the current qualitative meta-analysis, according to literature’s findings, provided evidence from 29 studies (see Clusters 3) that many well-siblings experience a lack of understanding of illness and of the needs of the brother or sister with MI (Brolin et al., 2024; Chen and Lukens, 2011; Friedrich et al., 2008; Heneghan et al., 2024; Hovland et al., 2025; Izon et al., 2024; Kinsella et al., 1996; Schaffer, 2021a). When confronted with the onset of MI in a brother or sister, well-siblings must first address the issue of causal attribution and interpretation to better accept their brother or sister’s MI. In this vein, and with significant cultural variations, several possible causal attributions are ranging from factors related to early family socialization, to genetic causes, to traumatic events that occurred later in life, to religious beliefs, supernatural causes, or fate (Chen et al., 2025; Hovland et al., 2025; Kawanishi, 2004; Mulipola et al., 2023; Tharoor and Ganesh, 2015). The process of causal attribution and interpretation of MI is an integral part of the process of adjustment and better acceptance of a sibling’s MI. However, in the experience of “navigating the storm” of MI, one of the most common initial reactions to the onset of MI in a sibling is denial (Muhlbauer, 2002). Differently, in gaining initial awareness, as indicated by Clark and Drake (1994), well-siblings must first recognize the existence of the problem (MI), despite the difficulty in defining and understanding it, which is usually accompanied by growing concern and often ineffective efforts to seek assistance. As awareness of a brother or sister’s MI progresses, factors such as worsening of the situation, initial contact with the mental health care system (often due to hospitalization), increased emotional distress, problems in interacting with health care professionals, and possible financial difficulties can come into play. A subsequent phase is characterized by the growing and painful realization that MI is a chronic condition and is often characterized by further emotional instability and recurring crises, feelings of anger, grief, and a sense of loss of the sibling as they knew them before the onset, a search for explanations, treatments and further and more in-depth knowledge, intensified financial concerns, dissatisfaction with health services (Schaffer, 2021b), and issues related to stigma (van der Sanden et al., 2015). At this point, it becomes necessary for the well-sibling to deal with necessary and substantial changes in their thoughts, values, and behaviors, such as finding ways to regain control, managing feelings of guilt and helplessness, changing perceptions and expectations, and finding ways to manage symptoms (Schaffer, 2021a), by creating workable care patterns and using a variety of support sources. From what has been said, it seems that it is only at the end of this long journey that, looking back, well-siblings could reach a stage characterized by subjective awareness of personal growth, in terms of gained meaning and value, but also by more realistic concerns for the future of their brother or sister with MI. Furthermore, the findings indicated that well-siblings experience a lack of support and challenges in accessing resources. Indeed, as indicated by literature findings, many well-siblings of people with mental illness feel that their experiences and needs go largely unnoticed by mental health services and often face barriers when attempting to engage with mental health systems, as clinical attention and treatment planning tend to focus almost exclusively on the individual with the diagnosis. As a result, well-siblings’ own emotional and practical needs are frequently overlooked, contributing to a heightened sense of burden and reduced access to appropriate support resources. On the contrary, literature findings agree in indicating that perceived social support serves as a buffer in reducing well-siblings’ distress. In particular, sharing their lived experience with friends, family members, or significant others may help well-siblings to reduce the levels of stress, anxiety, and depression (Brolin et al., 2024; Chen and Lukens, 2011; Hovland et al., 2025; Jayasinghe et al., 2023a, 2023b; Lecciso et al., 2025; Schaffer, 2021a; Schaffer, 2021b).

Although research on this topic has focused mainly on the burden of well-siblings of brothers or sisters with MI, it seems necessary to pay attention to and identify resilience factors that can help well-siblings in the process of adapting to the crisis that characterizes the discovery and subsequent gradual adjustment to the MI of a brother or sister. In this vein, the findings from the present qualitative meta-analysis (see Clusters 4) provided evidence from 27 studies that, despite the several significant challenges, the experience of having a brother or sister with MI, positive changes and resources are also reported. Indeed, posttraumatic growth (Szmukler et al., 1996) – considered as the result of caregiver experiences of struggling with challenging circumstances or events – emerged to consist also in personal growth, in terms of increased personal strengths and confidence, of more compassion, comprehension, tolerance, and empathy toward peoples in similar difficult situations, as well as of focusing on positive life experiences (Chen and Lukens, 2011; Dijkxhoorn et al., 2023; Heneghan et al., 2024; Kinsella et al., 1996; Leith et al., 2018; Sanders and Szymanski, 2013; Smith and Greenberg, 2008). The results also revealed that this last theme is strictly intertwined with positive changes in sibling relationships – primarily when characterized by greater reciprocity – and, more generally, family relationships – primarily when characterized by more open communication and mutual emotional and practical support (Dulek et al., 2021; Greeff et al., 2006; Bishop and Greeff, 2015; Heneghan et al., 2024; Smith et al., 2007). Furthermore, our findings, in line with previous studies, indicate that although well-siblings of individuals with MI are frequently exposed to complex and enduring emotional challenges, their experiences are not exclusively characterized by distress or hopelessness. Through processes of understanding, access to supportive resources, and ongoing personal development, they often cultivate a resilient, well-informed, and firmly grounded in reality hope for the future. This adaptive hope enables them to engage with the future not in the absence of fear, but with a strengthened sense of agency, purpose, and possibility (Bishop and Greeff, 2015; de Maynadier et al., 2015; Jensen et al., 2025).

Finally, the findings from the present qualitative meta-analysis (see Clusters 5) provided evidence from 34 studies on caregiving attitudes, experiences, and future expectations of well-siblings of people with MI. Indeed, well-siblings of individuals with MI often develop complex caregiving attitudes shaped by emotional ties, family dynamics, and societal pressures. While some feel a deep sense of duty to support their sibling with MI, others feel ambivalent due to the ongoing emotional, practical, and psychological burdens of caregiving. Early family roles, parental expectations, and cultural norms about family obligation often influence these feelings. Looking ahead, well-siblings commonly worry about long-term caregiving, especially as parents age, and uncertainty about the illness’s progression, service availability, and their own life paths. Many calls for greater recognition, education, and institutional help to manage their evolving roles while protecting their own well-being (Brolin et al., 2024; Dulek et al., 2021; Greenberg et al., 1999; Hatfield and Lefley, 2005; Leith et al., 2018; Phillips et al., 2023; Pierazzuoli et al., 2020; Smith et al., 2007; Stein et al., 2020; Wulandari et al., 2024). In this vein, when taking on the role of caregiver, well-siblings can go through different stages. Karp and Tanarugsachock (2000), for example, highlight a specific sequence, with a first stage, before diagnosis, in which the prevailing experience is one of emotional anomie. A second stage, coinciding with receiving the diagnosis and, therefore, an initial medical understanding of MI, arouses feelings of hope, compassion, and sympathy. Subsequently, the awareness that MI may be a permanent condition arouses feelings such as anger and resentment. Finally, awareness of and acceptance of their brother or sister’s MI’s uncontrollability allows well-siblings to reduce their involvement without feeling guilty. Another very significant area of study is that relating to the coping skills or strategies (such as problem-centered, emotion-centered, social support seeking, management of meaning, management of situation, management of distress, or distancing) used by well-siblings of people with MI (de Maynadier et al., 2015; Friedrich et al., 2008; Kinsella et al., 1996; Osuchowska-Kościjańska et al., 2014). These can range from physically or mentally distancing themselves from their family environment to find relief from the pressures of living with a sibling with MI, actively seeking support from others (such as friends, family members, mental health practitioners), objectifying the MI (differentiating between the mental illness and the person of brother or sister) (Schaffer, 2021b). It also emerges as very significant gathering information and knowledge about MI to better understand the brother or sister with MI and to reduce the sense of helplessness (Sporer et al., 2019), as well as relying on a form of spiritual faith or beliefs (Alfanta et al., 2024; Mulipola et al., 2023; Susanti et al., 2024), and escaping or avoiding an extremely painful emotional impact by internalizing strong negative emotions to reduce discomfort. Finally, other coping skills or strategies can range from recurring to drug use, monitoring and modifying one’s own behavior or adopting a specific family role as a means of controlling or circumventing stressful situations related to a sibling’s MI, avoiding social contact for fear of the stigma surrounding MI, to distancing himself from him, if not from the family as a whole.

### Limitations and strengths

As with all qualitative meta-analyses, the findings of this study reflect the quality of the original research included and examined. In line with Timulak’s (2009) recommendations for maintaining high-quality standards, we restricted inclusion to peer-reviewed journal articles. Consequently, we cannot determine whether unpublished studies would have yielded different results. Moreover, although the reviewed studies recruited participants from 11 countries, socio-political and socio-cultural factors shape how MI is experienced across contexts. Finally, the participants in the studies reviewed were well-siblings of individuals with specific MIs, including primarily schizophrenia, eating disorders, bipolar disorder, depression, and psychosis. For these reasons, caution is warranted when applying our findings to other contexts or pathology.

A key strength of this study lies in the use of multiple methodological integrity checks, including memoing, researcher consensus, and saturation, as well as quality assessment of reviewed studies (see the Method section). This qualitative meta-analysis aimed not only to describe the lived experiences of well-siblings of individuals with MI but also to examine the interplay between individual, familial, and social factors that influence these experiences. Because our results are based on the synthesis of independent studies, they offer reliable and robust accounts of the lived experiences of well-siblings of people with MI.

## Conclusion

Taken together, the findings of this qualitative meta-analysis examining the lived experiences of well-siblings of individuals with MI illustrate the multifaceted impact of having a brother or sister with MI. Well-siblings reported experiencing a wide range of challenges, including caregiving burden, stigma, psychological distress, reduced well-being, fear of contagion, parentification, and strained family relationships, among others. At the same time, positive outcomes were also identified, particularly in terms of personal growth. Given that the lived experience of well-siblings of people with MI remains a relatively underexplored and understudied area, this meta-analysis contributes to the advancement of the field by identifying areas that may be associated with an increased risk of siblings’ emotional difficulties, as well as critical directions for future research. In this regard, future research could focus on qualitative studies involving sibling pairs in which one of the siblings has an MI, studies involving well-siblings of siblings with a broader spectrum of MIs, studies examining gender and birth order and their influence on assuming caregiving roles, or longitudinal studies that could help understand how the relationship between well-siblings and a brother or sister with MI evolves over time. Indeed, a better understanding of the lived experience of siblings of peoples with MI could hold significant implications for mental health professionals who, by recognizing the unique challenges and emotional difficulties faced by this population, could develop tailored interventions and support services designed to address their specific needs. Moreover, by providing a deeper understanding of the complex and interconnected dynamics involved in caring for a sibling with MI, the findings underscore the need for targeted interventions that promote closer collaboration between well-siblings, families, and formal mental health services in both care provision and caregiver support. Moreover, strengthening connections with local communities through educational programs may help well-siblings navigate their caregiving roles, manage realistic expectations, and reduce stigma and burden. Finally, the development of policies that address well-sibling and family relationships, as well as the sociocultural factors shaping caregiving contexts, emerges as a crucial priority for improving positive outcomes not only for individuals with MI but also for their caregivers.

## Data Availability

The original contributions presented in the study are included in the article/supplementary material, further inquiries can be directed to the corresponding author.
